# *Doru luteipes* (Dermaptera: Forficulidae): Life History, Predatory Potential, and Rearing Method of Earwigs

**DOI:** 10.1007/s13744-026-01415-5

**Published:** 2026-07-22

**Authors:** Ana Luisa Rodrigues-Silva, Letícia Pereira Silva, Rosangela Cristina Marucci

**Affiliations:** Department of Entomology, Biological Control Laboratory, Federal University of Lavras (UFLA), Lavras, Minas Gerais Brazil

**Keywords:** Biological control, Natural enemy, Predation, *Spodoptera frugiperda*

## Abstract

The earwig *Doru luteipes* (Scudder) (Dermaptera: Forficulidae) is recognized as the most frequently encountered predatory insect species in maize agroecosystems across maize-producing regions of Brazil and other South American countries. For over three decades, the earwig has been extensively investigated through both field surveys and controlled laboratory studies aiming at biological control of insect pests. Although augmentative biological control strategies involving the release of *D. luteipes* have been proposed, the currently available data on its predatory behavior and efficiency remain scarce and scattered, hindering definition of its true potential as a biocontrol agent. In this review, we present a comprehensive synthesis of the current scientific knowledge on the life history of *D. luteipes*, highlighting studies that indicate the potential of the earwig in regulating populations of insect pests and fungal structures, including photographic records of its predatory activity. In addition, we propose a standardized and replicable rearing protocol for *D. luteipes*, which involves the use of acrylic straws as oviposition substrates and shelters, corrugated cardboard as a structural refuge, and the provision of a nutritionally adequate artificial diet for both nymphs and adults. The described method indicates to be suitable not only for rearing *D. luteipes* but also for other species within the order Dermaptera. Finally, we outline key research gaps and propose future directions for experimental and applied investigations targeting this natural enemy species, whose ecological role and full potential as a biological control agent in diverse agricultural systems remain insufficiently explored and understood.

## Introduction

Approximately 2200 species have been described in the Dermaptera order (Haas 2021). A significant part of this diversity, 117 species, was recorded in Brazil, occurring in several agricultural regions (Hopkins et al. 2022; Heleodoro 2024). Distributed in 12 families (Hopkins et al. 2022), earwigs are able to prey on a large number of insect pest species (Silva et al. 2022a; Orpet et al. 2025; Perrin et al. 2025; Paula et al. 2026; Gambari et al. 2026). Together with other macrobiological agents, earwigs play a prominent role in the natural suppression of crop pests (Rankin and Palmer 2009; Meunier 2024). Yet, they have still been the focus of few studies.

Dermapterans are generally nocturnal predators; during the day, they shelter in dark and humid places (Hehar et al. 2008; Campos et al. 2011) and practice predatory activity mostly at night, taking advantage of a space with minimal competition from diurnal predators (Naranjo-Guevara et al. 2017; Silva et al. 2023). Their thigmotactic habits enable them to encounter insect pests with cryptic behavior that are often difficult to manage through conventional control methods.

The most widely studied Dermaptera species in Brazil is *Doru luteipes* (Scudder) (Dermaptera: Forficulidae), mainly because it is found throughout Brazilian territory (Heleodoro 2024) and is easy to rear. It can be maintained in the laboratory for several generations, and large-scale production to supply biological control programs would be possible (Silva et al. 2022a). Although *D. luteipes* is frequently observed in maize *Zea mays* (Linnaeus) fields (Pasini et al. 2007), information on its predatory activity is limited and fragmentary, mainly based on laboratory tests, which may not represent its true potential.

We seek to address this gap through this review on the biological aspects of *D. luteipes* and its role as a predator of insect pests, highlighting the importance of its preservation. We also aim to present a large-scale rearing method, which could also be applied to other dermapterans, and indicate the direction of research to reveal the potential of the species as a biocontrol agent in tropical regions in Brazil and other countries where it occurs. The Google Scholar, Web of Science, and Scopus platforms were the main search tools used to prepare this review. The search focused on works published from 1988 to 2025 containing the keywords “Doru luteipes,” “Spodoptera frugiperda,” “Zea mays,” and “biological control.”

## Morphology and Biology

**Fig. 1:**
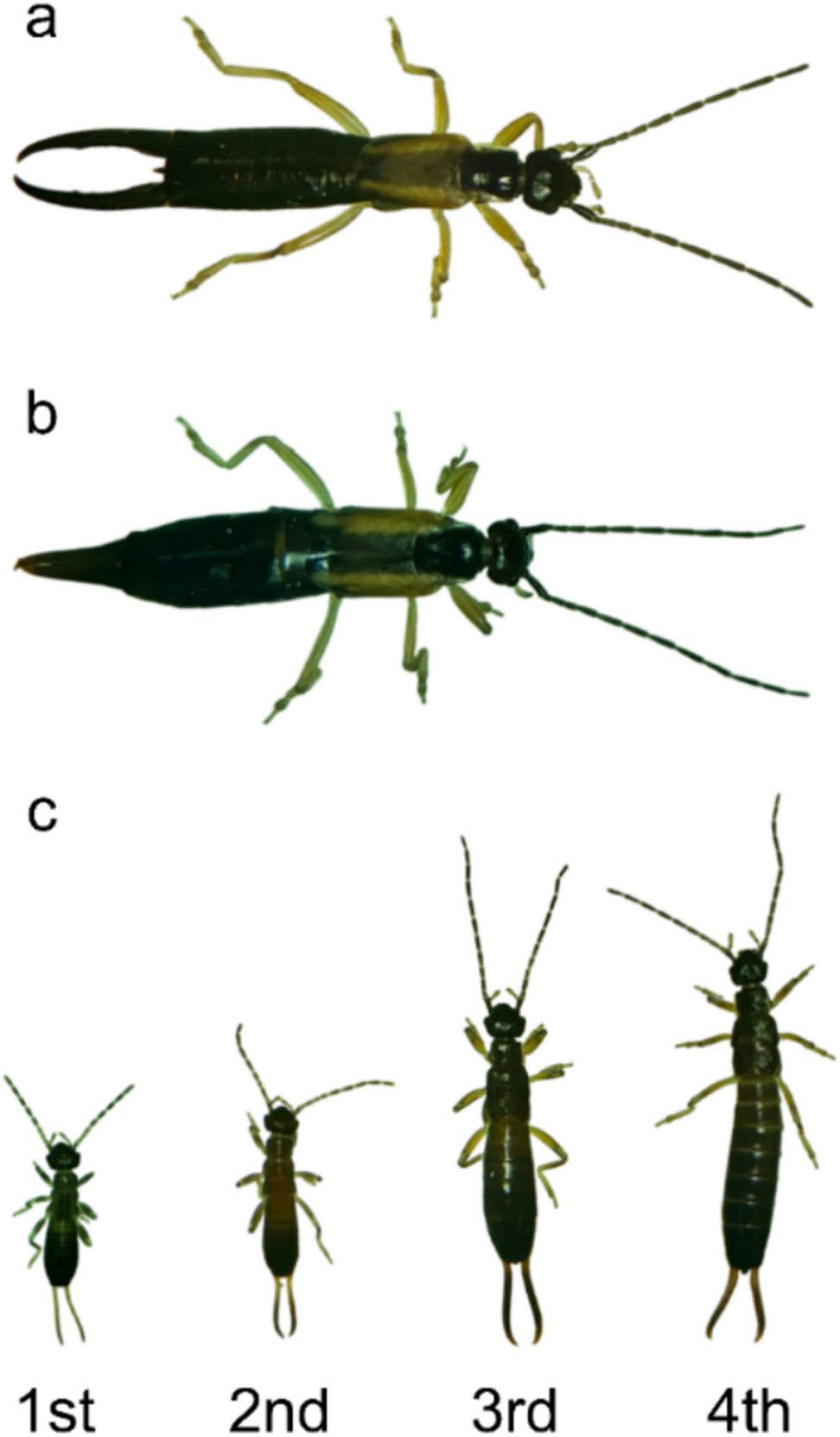
Male (**a**), female (**b**), and nymphal stages (**c**) of *Doru luteipes*

*Doru luteipes* has chewing mouthparts, three pairs of walking legs, brachyelytrous forewings, and terminal abdominal cerci (Haas 2018). Adults have a dark brown body, with yellowish legs and brachyelytrous; nymphs have a completely brown body. The species’ body length averages 15.5 mm, with males often larger than females. The differentiation between males and females (sexual dimorphism) is only apparent in adult insects; males have larger cerci that diverge and curve outward (Fig. 1a), whereas females have smaller, straighter cerci that remain closer together from base to apex (Fig. 1b).

**Fig. 2:**
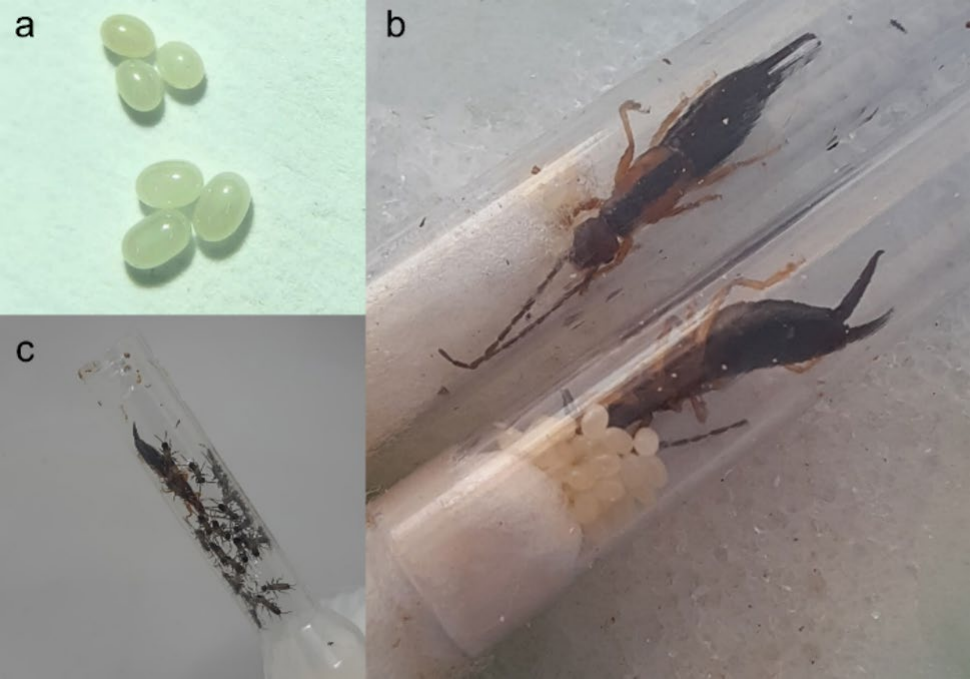
Difference in size of *Doru luteipes* eggs depending on the age of the eggs (**a**); *Doru luteipes* females tending eggs in the sheltered oviposition site (**b**); *Doru luteipes* female tending newly hatched nymphs (**c**) Source: Authors (2025)

Copulation occurs about 3 days after the female emerges, through a mating ritual (Silva and Marucci 2025), also observed in other Dermaptera species (Moore and Wilson 1993; Briceño and Eberhard 1995; Rankin et al 1995), in which the male dances, waving his cerci horizontally and approaching the female. The female can refuse the male by moving away or accept him by turning her cerci towards his, allowing the reproductive tracts to engage (Silva and Marucci 2025). The first egg clutch is laid approximately 15 days after emergence and the clutches are placed in moist, sheltered, and preferably dark sites (Silva and Marucci 2025). The eggs are whitish and increase in size as embryonic development progresses, allowing a view of nymphs’ eyes within (Fig. 2a). Females tend the clutch, cleaning the eggs daily and stacking them randomly (Fig. 2b). After 9 days, the nymphs hatch and remain under the female’s care for approximately 3 days (Silva and Marucci 2025) (Fig. 2c).

Maternal care in Dermaptera aims to protect eggs and nymphs against possible predators and the growth of opportunistic or entomopathogenic fungi (Jordan and Hefferan 1966; Boos et al. 2014; Greer et al. 2020; Meunier 2024; Silva and Marucci 2025). In *D. luteipes*, this behavior was associated with protection of offspring against two of the most widely studied, commercialized, and applied entomopathogenic fungi species, *Beauveria bassiana* (Balsamo-Crivelli) Vuillemin and *Metarhizium anisopliae* (Metchinikoff) Sorokin (Hypocreales: Clavicipitaceae) (Resende et al. 2025).

Following the maternal care period, the female takes about 12 days to lay her next clutch (Silva and Marucci 2025). The nymphs leave the sheltered oviposition site after maternal care and seek shelter in dark, narrow sites. Like adults, nymphs are omnivorous, feeding on both small arthropods and plant resources such as pollen. The four nymphal stages last an average of eight, six, seven, and four days, respectively, so the juvenile stage is complete in 30 days (Pasini et al. 2007). Instar differentiation is based on the development of the thoracic brachyelytra. First instar nymphs do not have brachyelytra, which will develop as the nymphal stage progresses (Fig. 1c).

## *Doru luteipes*: habit and prey

The earwig *D. luteipes*, the most abundant predatory insect in maize fields in Brazil (Cruz 1995a; Silva et al. 2018), is present in all major production regions (Reis et al. 1988; Marucci et al. 2019) during the summer and winter. It is observed on up to 70% of plants where maize is grown year-round (Picanço et al. 2003; Araújo et al. 2011; Silva et al. 2022a). The species has thigmotactic behavior and is stimulated by direct contact with conspecifics and plants (Jarvis et al. 2005; Pacheco et al. 2023). Although this earwig has already been observed in soybean *Glycine max* (L.) Merrill, sorghum *Sorghum bicolor* (L.) Moench, sugar cane *Saccharum officinarum* Linnaeus, cotton *Gossypium hirsutum* Linnaeus, and kale *Brassica oleracea* Linnaeus crops (Reis et al. 1988; Bacci et al. 2001, 2002; Fenoglio and Trumper 2007; Romero Sueldo et al. 2010), maize is considered its preferred habitat. The maize plant whorl, with tightly packed leaves, provides a high moisture microclimate and is an ideal site for protection, reproduction, and oviposition of the species. The nymphal stage of *D. luteipes* lasts approximately 30 days and adults can live for up to one year (Reis et al. 1988). Consequently, the species is able to remain in the field throughout its cycle, protecting the crop from potential arthropod pests.

As a generalist predator, *D. luteipes* has a diverse diet; nymphs and adults feed on aphids, thrips, and eggs and early larval instars of the order Lepidoptera (Fernandes et al. 2007; Pacheco et al. 2023; Silva et al. 2023). It is highly voracious, resulting in high predatory capacity (Romero Sueldo and Virla 2017), and omnivorous, feeding on pollen (Marucci et al. 2019) and fungal spores (Silva et al. 2022a).

Although earwigs species such as the European earwig *Forficula auricularia* Linnaeus (Dermaptera: Forficulidae) and the ring-legged earwig *Euborellia annulipes* (Lucas) (Dermaptera: Anisolabididae) have already been observed feeding on plant tissue and causing economic damage to crops (Kocarek et al. 2015; Orpet et al. 2019; Quarrell et al. 2021; Binns et al. 2022; Hanel et al. 2023, 2025; Nascimento et al. 2025), such behavior has not been observed for *D. luteipes* in any crop where it occurs. On the other hand, several authors have evaluated the potential of *D. luteipes* for predation or consumption of insect pest species and fungal structures (Table 1).

**Table 1:** Action of *Doru luteipes* on different prey and pathogen

Prey	Order	Family	Developmental stage	Location	Source
*Schizaphis graminum* (Rondani)	Hemiptera	Aphididae	Adults and nymphs	Brazil Field (sorghum)	Alvarenga et al. 1995 Alvarenga et al. 1996
*Brevicoryne brassicae* (Linnaeus)				Laboratory	Bacci et al. 2001
*Myzus persicae* (Sulzer)					Bacci et al. 2002
*Rhopalosiphum maidis* (Fitch)					Romero Sueldo et al. 2014 Marucci et al. 2019
*Aphis gossypii* Glover				Brazil Field (cotton) Laboratory	Sujii et al. 2007
*Dalbulus maidis* (DeLong and Wolcott)		Cicadellidae	Nymphs	Laboratory	Bortolotto et al. 2024
*Caliothrips phaseoli* (Hood)	Thysanoptera	Thripidae	Adults	Laboratory	Silva et al. 2022b
*Helicoverpa zea* (Boddie)	Lepidoptera	Noctuidae	Eggs	Laboratory	Cruz et al. 1995
*Helicoverpa armigera* (Hubner)					Ribeiro et al. 2017 Redoan et al. 2014
*Spodoptera frugiperda* (Smith)			Eggs and nymphs (1st to 3rd instar)	Brazil Field (maize) Laboratory	Reis et al. 1988 Araújo et al. 2011 Redoan et al. 2014 Naranjo-Guevara et al. 2017 Souza et al. 2021 Silva et al. 2023
*Diatraea saccharalis* (Fabricius)		Crambidae	Eggs	Argentina Field (maize) Laboratory	Fenoglio and Trumper 2007
*Plutella xylostella* (Linnaeus)		Plutellidae		Laboratory	Pedroso et al. 2010
*Ephestia kuehniella* (Zeller)		Pyralidae			Redoan et al. 2014
*Puccinia polysora* (Underw)	Pucciniales	Pucciniaceae		Brazil Field (maize) Laboratory	Silva et al. 2022a Pacheco et al. 2023

Studies have shown the role of *D. luteipes* as a biological control agent against aphid pests, including species that infest maize crops. This earwig is able to significantly reduce *Schizaphis graminum* (Rondani) (Hemiptera: Aphididae) populations on susceptible, moderately resistant, or highly resistant sorghum genotypes (Alvarenga et al. 1995, 1996). *Doru luteipes*, as well as coccinellid beetles, was identified as one of the most abundant predators of the aphid *Aphis gossypii* Glover (Hemiptera: Aphididae) in cotton-growing areas (Distrito Federal, Brazil) (Sujii et al. 2007). Romero Sueldo et al. (2014) found that *D. luteipes* and *Doru lineare* (Eschscholz) (Dermaptera: Forficulidae) are important predators, with similar potential in reducing populations of the maize aphid *Rhopalosiphum maidis* (Fitch) (Hemiptera: Aphididae) (Tucumán, Argentina), which causes direct and indirect damage to maize by feeding on the crop and transmitting plant viruses.

Marucci et al. (2019) also evaluated the feeding behavior of *D. luteipes* on *R. maidis* in the laboratory and found that the predator survived for a shorter time when fed exclusively with the aphid, as that prolonged nymphal development and increased nymph mortality. Consequently, this and other aphid species may not provide all the nutrients necessary for full development of *D. luteipes* under field conditions, making maize pollen an important supplement. In the same study, a diet exclusively of *Spodoptera frugiperda* (Smith) (Lepidoptera: Noctuidae) eggs likewise did not favor full development of the predator.

*Doru luteipes* has been reported to prey on other sap-sucking insects, including nymphs of the corn leafhopper *Dalbulus maidis* (DeLong and Wolcott) (Hemiptera: Cicadellidae) (Bortolotto et al. 2024). Silva et al. (2023) studied the predation of *D. luteipes* on *Caliothrips phaseoli* (Hood) (Thysanoptera: Thripidae), an important common bean and soybean pest (Monteiro et al. 1999). Adults of *D. luteipes* consumed 210 thrips per day, a consumption rate approximately six times greater than that achieved by *Orius insidiosus* (Say) (Hemiptera: Anthocoridae), a predatory stink bug sold in several countries for control of thrips. Even first-instar *D. luteipes* nymphs consumed more thrips than *O. insidiosus* adults. The authors suggested that the thigmotactic behavior of *D. luteipes* would enable it to occupy the same niche as several species of thrips, which are characterized by their cryptic habit. Furthermore, the earwig would complement the control provided during the day by *O. insidiosus*, as it feeds exclusively at night. No intraguild predation was observed between the earwig and the predatory stink bug.

**Fig. 3:**
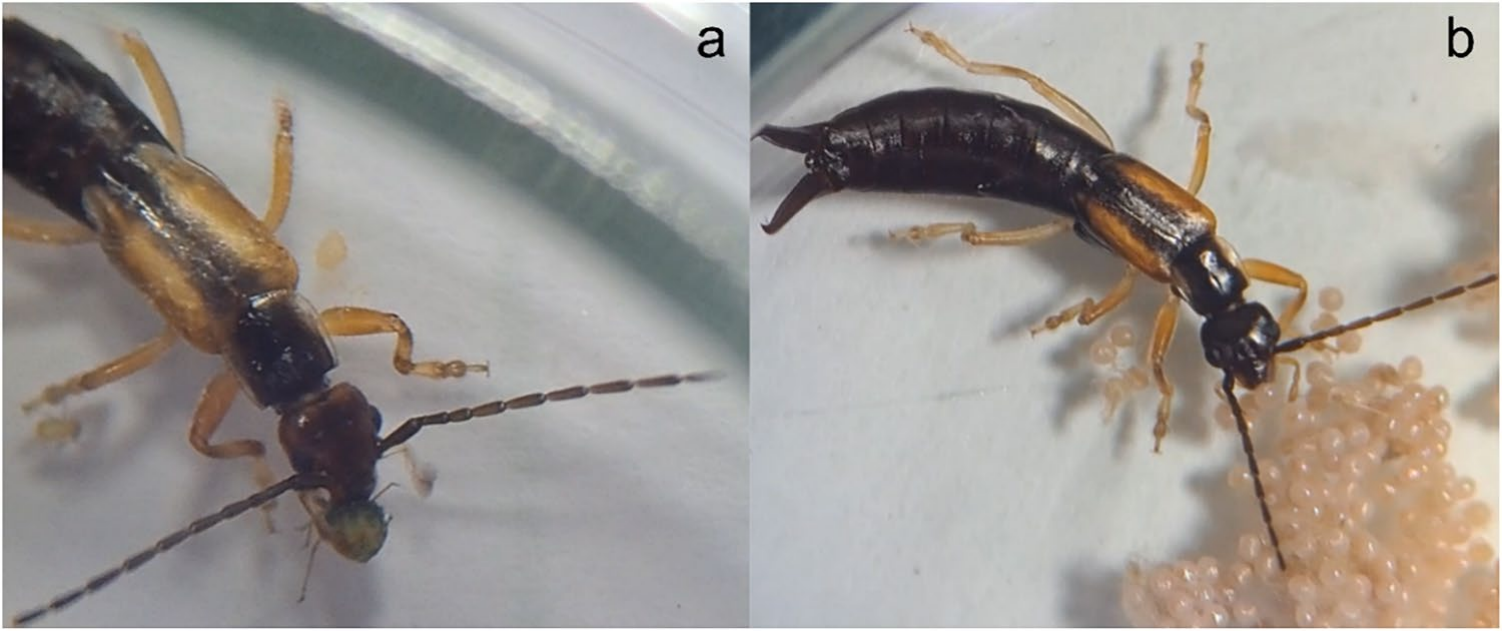
*Doru luteipes* adult feeding on the aphid *Rhopalosiphum padi* (**a**) and *Spodoptera frugiperda* eggs (**b**) Source: Authors (2025)

A good number of studies have evaluated the role of *D. luteipes* in control of aphid species (Fig. 3a). However, the earwig’s potential for locating these sap-sucking insects and reducing their populations has not yet been deeply investigated. The predominant aim of studies was to evaluate the selectivity of insecticides, and they did not quantify the predatory activity of *D. luteipes* (Bacci et al. 2001, 2002). Therefore, the regulatory effect of *D. luteipes* on species of aphids and/or thrips under field conditions is still unknown.

This earwig is considered an important and indispensable natural enemy in managing populations of lepidopterans that defoliate maize crops, such as the fall armyworm *S. frugiperda* and the earworm *Helicoverpa zea* (Boddie) (Lepidoptera: Noctuidae) (Reis et al. 1988; Cruz 1995a). Adults and nymphs of the earwig can be found in whorls, tassels, and ears of maize plants, allowing the predator to find prey and act (Marucci et al. 2019). Furthermore, *D. luteipes* is not negatively affected by maize hybrids with *Bacillus thuringiensis* (Bt) (Berliner) (Bacillales: Bacillaceae) genes, and it can complement the control of caterpillars provided by this technology (Fernandes et al. 2007; Araújo et al. 2011).

*Spodoptera frugiperda* is a key pest of maize and one of the main factors that limit the development and yield of the crop (Oliveira et al. 2014; Nagoshi et al. 2015). It causes damage at all stages of development, leading to significant yield losses, from 17 to 39% (Cruz 1995a; Silva et al. 2023). The greatest damage to the crop occurs after formation of the whorl, where the caterpillar takes shelter, as the pest can damage the apical meristem, potentially killing the plant (Araújo et al. 2011). Furthermore, inside the whorl, the insect is protected from insecticides, limiting the effectiveness of one of the main pest management methods (Araújo et al. 2011; Paredes-Sánchez et al. 2021). *Doru luteipes* protects maize from fall armyworm by preying on eggs and neonate caterpillars, and thus, a negative correlation has been observed between the presence of the natural enemy and the pest (Guerreiro et al. 2003; Badji et al. 2004). In fact, at least a pair of *D. luteipes* individuals in plants are known to significantly reduce caterpillar populations (Cruz 1991). Due to the large number of studies that discuss the role of *D. luteipes* in combatting *S. frugiperda*, the interaction between these two species will be addressed separately.

The biology and predatory capacity of *D. luteipes* fed on *H. zea* eggs have also been extensively studied. The earwig consumed an average of 8276 eggs throughout its cycle, an average of 39 eggs per day (Cruz 1995b). When earwigs fed exclusively on *H. zea* eggs, their life cycle was approximately 217.9 days (Cruz 1995b), much longer than the 128-day cycle observed when they fed on *S. frugiperda* eggs (Reis et al. 1988).

The feeding behavior of *D. luteipes* on *Helicoverpa armigera* (Hubner) (Lepidoptera: Noctuidae), *Plutella xylostella* (Linnaeus) (Lepidoptera: Plutellidae), and *Diatraea saccharalis* (Fabricius) (Lepidoptera: Crambidae) has also been evaluated. The average daily consumption of *H. armigera* eggs and newly hatched caterpillars was 16.8 and 12.7, respectively (Ribeiro et al. 2017). In contrast, in free-choice consumption, *D. luteipes* preferred to feed on *S. frugiperda* eggs, followed by those of *H. armigera* and *Ephestia kuehniella* (Zeller) (Lepidoptera: Pyralidae) (Redoan et al. 2014). *Doru luteipes* nymphs consumed a large number of *P. xylostella* eggs, on average of 4600, highlighting the voracity of the 27-day nymphal stage (Pedroso et al. 2010). In maize crops, the presence of *D. luteipes* was also positively correlated with the mortality of eggs of the sugarcane borer *D. saccharalis*. Upon distributing *D. saccharalis* sentinel eggs in the field (Córdoba, Argentina), Fenoglio and Trumper (2007) observed that *D. luteipes* consistently preyed on eggs throughout vegetative development of the crop, although predation declined in periods of intense rainfall.

## Potential of *Doru luteipes *in Regulating *Spodoptera frugiperda*

A field study to evaluate the performance of *D. luteipes* as a potential predator of *S. frugiperda* was developed by Reis et al. (1988). Earwig nymphs consumed an average of 465 newly hatched caterpillars, while adults consumed 2349 caterpillars. The average longevity of adults fed exclusively on caterpillars was 113.7 days, which did not differ significantly from the 83.2 days of adults fed exclusively on *S. frugiperda* eggs (Fig. 3b). However, these results contrast with those of Silva et al. (2022b), who observed a longevity of just 44.6 days for *D. luteipes* fed on neonates of *S. frugiperda*.

Araújo et al. (2011) evaluated the population dynamics of *D. luteipes* and *S. frugiperda* in conventional and Bt maize crops and found no significant differences (Goiás, Brazil). They concluded that the earwig is not negatively affected by Bt technology and that it contributes to suppressing population growth of the pest. Fifth-instar earwig nymphs are able to consume 70 eggs and 43 neonates of *S. frugiperda* in 24 h, and their predatory capacity and foraging behavior do not change when they feed on caterpillars resistant to the Cry1F protein (Souza et al. 2021).

Previous studies have shown that *S. frugiperda* females are able to differentiate intact maize plants from plants that have undergone herbivory, showing a preference for intact plants (Signoretti et al. 2012). The moths effectively identify the volatile compounds of plants that have been attacked and avoid intraspecific competition, laying eggs on non-infested plants (Pinto-Zevallos et al. 2016). Nevertheless, it is known that plants have evolved protective mechanisms, induced by herbivory, such as an increase in the concentration of secondary metabolites with toxic or deterrent effects on insects and the emission of volatile organic compounds (Mithöfer and Boland 2012; Pinto-Zevallos et al. 2016). These volatiles attract the natural enemies of herbivorous insects, recruiting them to the site of herbivory and promoting biological control (McCormick et al. 2012). Maize plants attacked by caterpillars of *S. frugiperda* and *Spodoptera littoralis* (Boisduval) (Lepidoptera: Noctuidae) release several terpenoids, green leaf volatiles, and indole, compounds that are detected by antagonistic insects and by the phytophagous insects themselves (Degen et al. 2004; De Lange et al. 2016; Pinto-Zevallos et al. 2016).

Naranjo-Guevara et al. (2017) found that *D. luteipes* is able to detect volatiles released by maize plants attacked by *S. frugiperda* and *D. saccharalis*, allowing it to locate its prey even at night. The attraction was greater toward plants that had been recently damaged (1–3 h). The researchers highlighted that even after a 48-h fasting period, the earwig was able to locate and feed on *S. frugiperda* exclusively at night. *Doru luteipes* is also attracted to volatiles emitted by arugula *Eruca sativa* (Brassicaceae) plants damaged by *S. frugiperda* (Bell et al. 2020).

In tests with free choice between eggs and neonate caterpillars of *S. frugiperda*, *D. luteipes* showed a preference for consuming eggs, which could prevent injuries from occurring in the field (Rodrigues‐Silva et al. 2024). However, *D. luteipes* nymphs fed exclusively on *S. frugiperda* eggs had an extended nymphal phase and a higher mortality rate (Marucci et al. 2019). Therefore, feeding exclusively on eggs does not ensure the nutrients necessary for full development of the species. It is known that supplementing the earwig’s diet with maize pollen allows it to survive under conditions of low prey density (Silva et al. 2022a) and improves survival and fertility rates (Marucci et al. 2019). In Brazil, *D. luteipes* is found throughout the year at all stages of maize vegetative development, when *S. frugiperda* is highly prevalent (Marucci et al. 2019). However, peak population of the predator coincides with the reproductive stage of the crop, as pollen plays an essential role in attracting and maintaining earwigs in the field (Maggio et al. 2022; Pacheco et al. 2023). This dynamic can delay suppression of early generations of the pest.

Due to the risk of predation, mated *S. frugiperda* females oviposit less on plants where earwigs are present (Rodrigues‐Silva et al. 2024). Additionally, *D. luteipes* adults do not feed on *S. frugiperda* caterpillars from the fourth instar onwards, due to the greater rigidity of the exoskeleton (Silva et al. 2022b). Therefore, strategies to promote earlier arrival of the predator must be taken so that earwigs reach the crop when maize is more susceptible to the pest (Maggio et al. 2022). Earlier earwig arrival would also help reduce the size of *S. frugiperda* egg clutches and allow earwig to find eggs and neonate caterpillars, avoiding injuries. The use of cover crops that produce large amounts of pollen during the maize off-season is a strategy that could allow early attraction of *D. luteipes*. That way, this biocontrol agent would already be in the field when the crop is established and could act on the first generations of *S. frugiperda*. However, greater knowledge is necessary in relation to plant species with potential for recruiting *D. luteipes*.

## *Doru luteipes*: Fungivory, Omnivory, and Survival

**Fig. 4:**
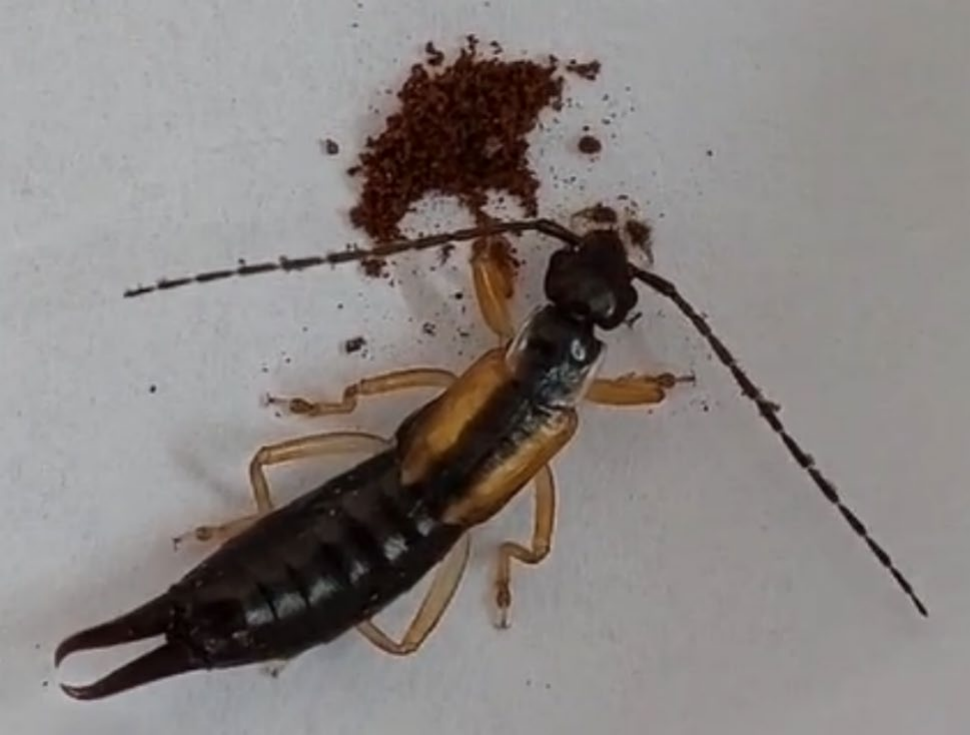
Female *Doru luteipes* feeding on uredospores of *Puccinia* sp. Source: Authors (2025)

A new ecosystem service performed by *D. luteipes* on maize plants was recently discovered. Silva et al. (2022a) reported that the earwig feeds on uredospores of *Puccinia polysora* (Underw) (Pucciniales: Pucciniaceae), the causal agent of corn polysora rust (Fig. 4). Both nymphs and adults fed on and reduced the concentration of uredospores in the laboratory and on infected plants in a greenhouse. The fungivory of *D. luteipes* was confirmed by evaluating the survival of newly hatched nymphs fed exclusively on *P. polysora* uredospores, maize pollen, *S. frugiperda* eggs, or a combination of *S. frugiperda* eggs and uredospores. The survival rate declined when nymphs received a single food source, but feeding on a combination of eggs and uredospores resulted in survival rates similar to those in the control treatment (artificial diet). This suggests that omnivory is essential for the survival and persistence of *D. luteipes* in the field (Pacheco et al. 2023), expanding its potential as a biocontrol agent against fungal diseases.

Methodologies based on molecular biology techniques have made it possible to identify the DNA of *S. frugiperda* and other arthropod pests in the contents of the digestive tract of natural enemies, including dermapterans (Maggio et al. 2022; Paula et al. 2022). These new technologies enable studies to characterize trophic interactions and understand the dynamics of *D. luteipes* in the field. Such research could clarify its actual role and impact in biocontrol of pests and/or pathogens.

## Laboratory Rearing

### Environmental Conditions

Populations are reared in the laboratory using insects previously collected from the field, mainly from maize crops at flowering time. The insects are then quarantined to eliminate potential contaminants. In the laboratory, insects should be kept in a controlled environment, with an average temperature of 26 ± 1 °C, relative humidity of 70 ± 10%, and a photoperiod of 14/10 h (light:dark).

### Stock Creation

**Fig. 5:**
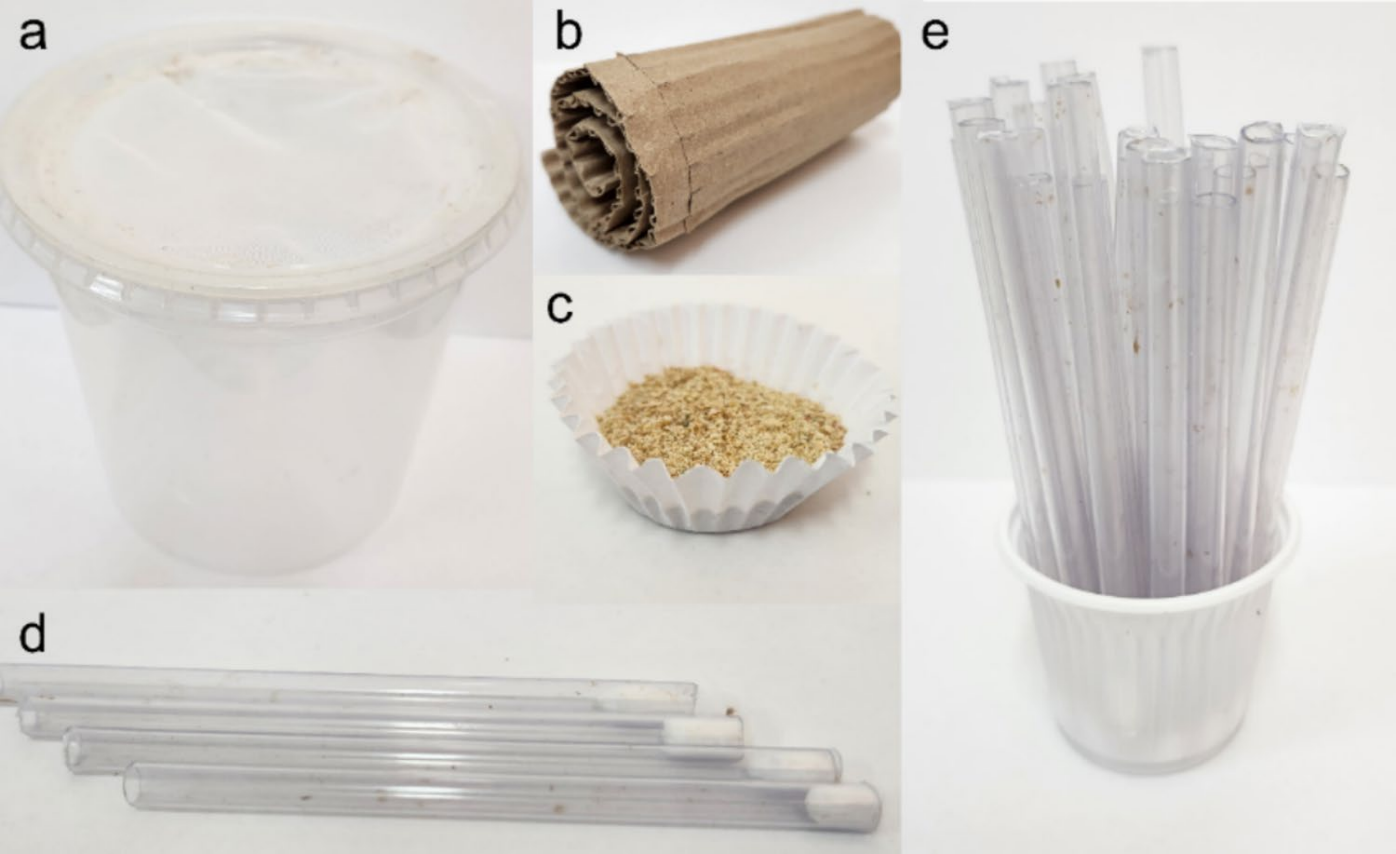
Composition of *Doru luteipes* rearing container. Plastic container with lid modified by cutting out a section and filling it with voile fabric (**a**); shelter composed of corrugated paper (**b**); artificial diet in paper cup (**c**); acrylic straws with cotton inserted in one end (**d**); oviposition shelters kept in a plastic cup with moistened cotton (**e**) Source: Authors (2025)

The earwigs are reared in plastic containers (12.5 × 14.5 cm) with the lid modified by cutting out a section and filling it with voile fabric, which prevents the escape of first instar nymphs (Fig. 5a). To support thigmotactic behavior, it is recommended to provide shelters such as folded or corrugated paper rolls (10 × 5 cm) (Fig. 5b) that allow insects to enter and exit easily. The artificial diet of nymphs and adults is composed of 35% cat food, 27% wheat bran, 23% brewer’s yeast, 14% powdered milk, 0.5% nipagin, and 0.5% sorbic acid (Cruz 2009). All ingredients are blended into a fine powder and provided ad libitum in paper cups (Fig. 5c). Providing the diet in paper containers is important as they allow insects to climb and reach the diet easily. The diet should be replaced weekly or as needed.

*Doru luteipes* benefits from a well-established artificial diet that is easy to prepare, has a long shelf life under refrigerated conditions, and presents a favorable cost–benefit ratio (Parra and Coelho 2022). In addition, the diet is provided as a dry powder which reduces the risk of fungal contaminations. Moreover, when this diet is provided, live prey is not required for the complete development of this species under laboratory conditions (Marucci et al. 2019; Pacheco et al. 2023; Silva and Marucci 2025). Nevertheless, the occasional provision of live prey, such as aphids, may be recommended to supplement the diet, particularly to support optimal nymph development and reduce the occurrence of malformations in adults.

To enable females to lay eggs, an oviposition shelter must be provided (Meunier 2024). The shelter is made from acrylic straws cut in half. Each half of the straw serves as an oviposition shelter, and for that purpose, a piece of cotton is inserted in one end (Fig. 5d). These oviposition shelters are then placed in 50-ml plastic cups, with the cotton-filled end facing downwards. Water is added to the cups to ensure the humidity required for embryonic development of the eggs (Fig. 5e).

**Fig. 6:**
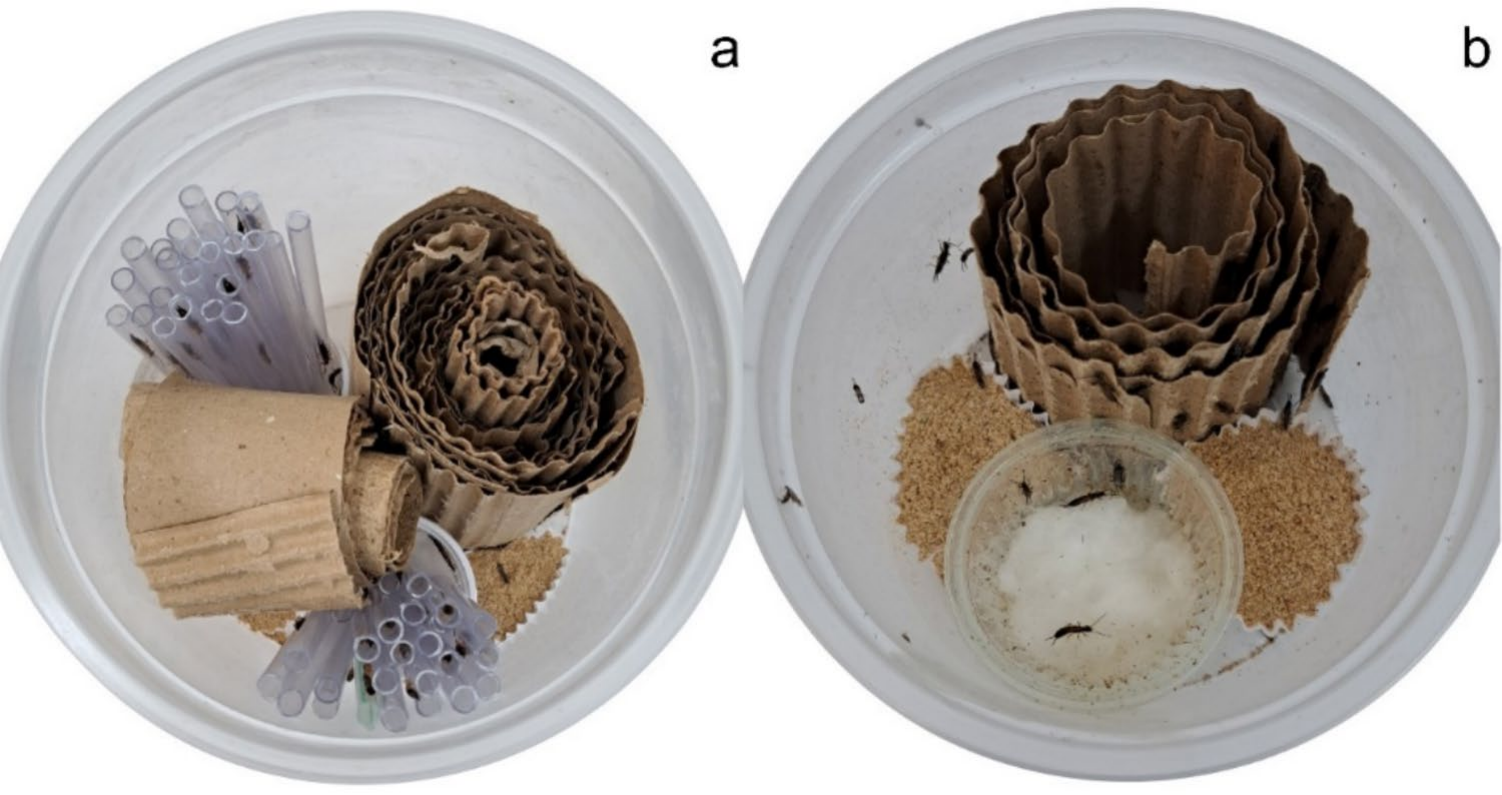
Rearing container for adults (**a**) and nymphs (**b**) of *Doru luteipes* Source: Authors (2025)

The adult rearing container consists of corrugated paper as a structural refuge, an artificial diet, and acrylic straws as oviposition shelters (Fig. 6a). In the nymph container, oviposition shelters are not necessary and are replaced by a moistened cotton pad (Fig. 6b).

### Rearing

To maintain the population at an adequate level, it is necessary to separate the egg clutches from the adult containers once a week by removing the oviposition shelters and transferring them to a new rearing cage. It is recommended not to exceed 25 egg clutches per container. Approximately 10 days after the nymphs hatch and leave the oviposition shelters, the females are returned to the adult containers, and the oviposition shelters are removed. The oviposition shelters are cleaned by discarding the cotton using tweezers and sanitizing the straws in a sodium hypochlorite aqueous solution (2%) for approximately 1 h, at which time they should be rinsed and air dried. The nymphs are kept in rearing containers until they develop into adults, with the diet and water replaced twice a week.

Approximately 40 days after the egg clutches are separated, adults begin to emerge, though this is not uniform and may extend for up to 2 weeks. Therefore, it is recommended to remove the newly emerged adults once a week and transfer them to new rearing containers containing oviposition shelters for the mated females. Each cage should contain at most 50 females, with at least one oviposition shelter per female. Water and diet should be replaced twice a week.

To increase the population and obtain a larger number of individuals for bioassays or for release, this same procedure should be carried out more frequently, removing the egg clutches and separating newly emerged adults twice a week. It is important to periodically inspect insects for any abnormalities.

### Case Study

**Fig. 7:**
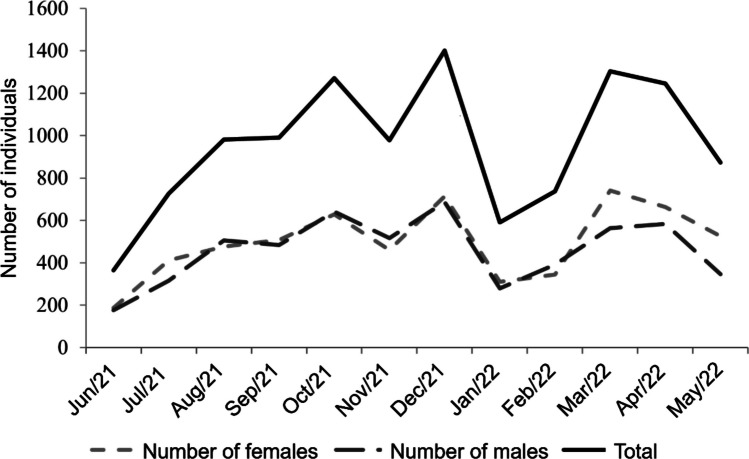
Obtaining *Doru* luteipes adults for experimental purposes from June 2021 to November 2022 using the proposed rearing methodology. Source: Authors (2025)

From June 2021 to November 2022, this methodology was developed at the Biological Pest Control Laboratory (LCBIOL) of the Department of Entomology, School of Agricultural Sciences (ESAL) of the Federal University of Lavras (UFLA). From June to December 2021, a total of 1401 individuals were obtained, 51% females and 49% males. After this period, the population size gradually declined. Over approximately 18 months, 13,948 *D. luteipes* adults were obtained, comprising 52.5% females and 47.5% males (Fig. 7).

During the maintenance of this earwig, the average monthly cost was R$23.42 (approximately US$4.70) for the artificial diet and R$23.56 (approximately US$4.73) for cotton, resulting in a total monthly cost of R$46.98 (approximately US$9.43) per month. Other materials (e.g., plastic containers, corrugated paper, acrylic straws), as well as permanent equipment (e.g., air conditioner, refrigerator), were not included in the calculation, as these items are reusable and would only represent an initial investment when establishing the rearing. Electricity costs were also not included, as the LCBIOL supports multiple activities and insect rearings in addition to *D. luteipes*.

Considering that a total of 13,948 *D. luteipes* adults were produced over an 18-month period, the estimated production cost was approximately R$0.06 (≈ US$0.01) per adult. This estimate highlights the relatively low cost of maintaining a laboratory rearing using the proposed methodology.

### Final Perspectives

Through this review article, we compile and organize relevant information about *D. luteipes* with the aim of facilitating access to existing knowledge for future researchers and supporting the advancement of new investigations. The potential of this earwig as a biological control agent of agricultural pests is undeniable; nevertheless, significant knowledge gaps remain to be addressed to enable the full exploitation of this entomological resource. Accordingly, we sought to highlight throughout the text the main points that require further investigation and to suggest possible directions for future studies.

Several important questions remain unresolved, including the mechanisms by which maternal care protects eggs and nymphs against pathogens. Although numerous studies have addressed the role of *D. luteipes* as biocontrol agent, its capacity to locate prey and regulate populations under field conditions remains poorly understood. Likewise, strategies capable of promoting earlier colonization of maize fields by earwigs, such as the use of cover crops, require further investigation, including the identification of suitable plant species. In addition, emerging molecular tools offer new opportunities to characterize trophic interactions and quantify the actual contribution of *D. luteipes* to pest suppression in agroecosystems. These gaps range from fundamental aspects of biology and behavior to applied strategies for use in integrated pest management programs, underscoring the broad scope for scientific and technological progress involving this species.

To encourage and support the continuation of research in this area, we also propose a standardized laboratory rearing methodology. This approach offers important advantages, as it requires low financial investment, is easy to implement, and efficiently reproduces the environmental conditions essential for the development and reproduction of *D. luteipes*. A distinctive feature of this methodology is the elimination of the need to maintain host plants, which considerably simplifies the process and broadens its applicability across different experimental contexts.

In the LCBIOL, this methodology has also been successfully applied for the rearing of *E. annulipes*, with minor modifications to accommodate its specific biological characteristics. Due to *E. annulipes* terrestrial habits and limited ability to climb smooth surfaces, it is recommended to arrange the oviposition shelters horizontally rather than vertically, thereby facilitating access and use by the insect. We therefore encourage the adaptation and validation of the proposed methodology for the mass rearing of other earwig species, always considering the biological and behavioral particularities of each one, in order to expand the use of these important natural agents in the sustainable management of agricultural pests.

## Conclusions

The results of studies on *D. luteipes* highlight its association with maize plants, which can shelter this voracious, long-lived, and cryptic predator with potential for regulating the population of aphids, thrips, and eggs and neonates of Lepidoptera. Maize also provides fungal structures and, in the absence of prey, offers pollen as a supplementary food. In tropical agriculture, maintaining generalist and omnivorous predators is a key strategy in dealing with high pest pressure and outbreaks, and it must be prioritized. Furthermore, the rearing methodology proposed for obtaining *D. luteipes* over several generations is viable for use in biological control programs involving multiple insect pests and pathogenic fungi.

## Data Availability

The datasets generated during and/or analyzed during the current study are available from the corresponding author on reasonable request.

## References

[CR1] Alvarenga CD, Vendramim JD, Cruz I (1995) Biologia e predação de *Doru luteipes* (Scud.) sobre *Schizaphis graminum* (Rond.) criado em diferentes genótipos de sorgo. An Soc Entomol Bras 24:523–531

[CR2] Alvarenga CD, Vendramim JD, Cruz I (1996) Efeito do predador *Doru luteipes* (Scud.) sobre o crescimento populacional de *Schizaphis graminum* (Rond.) em diferentes genótipos de sorgo. An Soc Entomol Bras 25:137–140

[CR3] Araújo LF, Silva AG, Cruz I et al (2011) Flutuação Populacional de *Spodoptera frugiperda* (J. E. Smith), *Diatraea saccharalis* (Fabricius) e *Doru luteipes* (Scudder) em Milho Convencional e Transgênico BT. Rev Bras Milho Sorgo 10:205–214. 10.18512/1980-6477/rbms.v10n3p205-214

[CR4] Bacci L, Picanço MC, Gusmão MR et al (2001) Seletividade de inseticidas a *Brevicoryne brassicae* (L.) (Hemiptera: Aphididae) e ao predador *Doru luteipes* (Scudder) (Dermaptera: Forficulidae). Neotrop Entomol 30:707–713

[CR5] Bacci L, Picanço MC, Gusmão MR et al (2002) Inseticidas seletivos à tesourinha *Doru luteipes* (Scudder) utilizados no controle do pulgão verde em brássicas. Hortic Bras 20:174–179. 10.1590/S0102-05362002000200011

[CR6] Badji CA, Guedes RNC, Silva AA, Araújo RA (2004) Impact of deltamethrin on arthropods in maize under conventional and no-tillage cultivation. Crop Prot 23:1031–1039. 10.1016/j.cropro.2004.03.003

[CR7] Bell K, Naranjo-Guevara N, Dos Santos RC et al (2020) Predatory earwigs are attracted by herbivore-induced plant volatiles linked with plant growth-promoting rhizobacteria. InSects 11:271–282. 10.3390/insects11050271 32365691 10.3390/insects11050271 PMC7290886

[CR8] Binns MR, Macfadyen S, Umina PA (2022) The dual role of earwigs (Dermaptera) in winter grain crops in Australia. J Appl Entomol 146:272–283. 10.1111/jen.12959

[CR9] Boos S, Meunier J, Pichon S, Kölliker M (2014) Maternal care provides antifungal protection to eggs in the European earwig. Behav Ecol 25:754–761. 10.1093/beheco/aru046

[CR10] Bortolotto OC, Santos JVC, Ochonski AG (2024) First report of earwig *Doru luteipes* (Dermaptera: Forficulidae) preying on corn leafhopper *Dalbulus maidis* (Hemiptera: Cicadellidae) nymphs. Braz J Biol 84:1–2. 10.1590/1519-6984.285975 10.1590/1519-6984.285975 39442152

[CR11] Briceño RD, Eberhard WG (1995) The functional morphology of male cerci and associated characters in 13 species of tropical earwigs (Dermaptera: Forficulidae, Labiidae, Carcinophoridae, Pygidicranidae). Smithson Contrib Zool 1–63:1. 10.5479/si.00810282.555

[CR12] Campos MR, Picanço MC, Martins JC et al (2011) Insecticide selectivity and behavioral response of the earwig *Doru luteipes*. Crop Prot 30:1535–1540. 10.1016/j.cropro.2011.08.013

[CR13] Cruz I (1991) Potencial de *Doru luteipes* como predador de *Spodoptera frugiperda* em condições de campo. Relatório Técnico Anual Do Centro Nacional De Pesquisa De Milho e Sorgo 4:85–86

[CR14] Cruz I (1995) Manejo integrado de pragas de milho com ênfase para o controle biológico. An Soc Entomol Brasil 24:48–92

[CR15] Cruz I (1995) A lagarta-do-cartucho na cultura do milho. EMBRAPA-CNPMS Circular técnica 21, Sete Lagoas, pp 1–45

[CR16] Cruz I (2000) Métodos de criação de agentes entomófagos de *Spodoperda frugiperda*. In: Bueno VHP (ed) Controle biológico de pragas: produção massal e controle de qualidade, 1st edn. Editora UFLA, Lavras, pp 111–135

[CR17] Cruz I, Alvarenga CD, Figueiredo PEF (1995) Biologia de *Doru luteipes* (Scudder) e sua capacidade predatória de ovos de *Helicoverpa zea* (Boddie). Anais da Sociedade Entomológica do Brasil 24:273–278. 10.37486/0301-8059.v24i2.1027

[CR18] Silva DD da, Mendes SM, Parreira DF et al (2022a) Fungivory: a new and complex ecological function of Doru luteipes (Scudder) (Dermaptera: Forficulidae). Braz J Biol 82.10.1590/1519-6984.238763 10.1590/1519-6984.238763 33825760

[CR19] da Silva HEG, de Brito CH, de Oliveira R (2022) Biological aspects and predatory capacity of *Doru luteipes* when fed with *Spodoptera frugiperda*. Rev Caatinga 35:490–497. 10.1590/1983-21252022v35n224rc

[CR20] Paula DcDSP, Barros SKA, Pitta RM et al (2022) Metabarcoding versus mapping unassembled shotgun reads for identification of prey consumed by arthropod epigeal predators. Gigascience 11:giac020. 10.1093/gigascience/giac020 10.1093/gigascience/giac020 PMC8952265 35333301

[CR21] De Lange ES, Farnier K, Gaudillat B, Turlings TCJ (2016) Comparing the attraction of two parasitoids to herbivore-induced volatiles of maize and its wild ancestors, the teosintes. Chemoecology 26:33–44. 10.1007/s00049-015-0205-6

[CR22] Degen T, Dillmann C, Marion-Poll F, Turlings TCJ (2004) High genetic variability of herbivore-induced volatile emission within a broad range of maize inbred lines. Plant Physiol 135:1928–1938. 10.1104/pp.104.039891 15299140 10.1104/pp.104.039891 PMC520764

[CR23] Fenoglio MS, Trumper EV (2007) Influence of weather conditions and density of *Doru luteipes* (Dermaptera: Forficulidae) on *Diatraea saccharalis* (Lepidoptera: Crambidae) egg mortality. Environ Entomol 36:1159–1165. 10.1603/0046-225x(2007)36[1159:iowcad]2.0.co;2 18284741 10.1603/0046-225x(2007)36[1159:iowcad]2.0.co;2

[CR24] Fernandes OA, Faria M, Martinelli S et al (2007) Short-term assessment of bt maize on non-target arthropods in Brazil. Sci Agric 64:249–255. 10.1590/S0103-90162007000300006

[CR25] Gambari LI, Duru PT, Singham GV, Salim H (2026) Sexual dimorphism in predatory efficiency of earwig *Chelisoches annulatus*, (Dermaptera: Chelisochidae) on bunch moth *Tirathaba rufivena* (Lepidoptera: Pyralidae). Zoomorphologie 145:4. 10.1007/s00435-025-00760-0

[CR26] Greer JA, Swei A, Vredenburg VT, Zink AG (2020) Parental care alters the egg microbiome of maritime earwigs. Microb Ecol 80:920–934. 10.1007/s00248-020-01558-x 32767092 10.1007/s00248-020-01558-x

[CR27] Guerreiro JC, Filho EB, Busoli AC (2003) Ocorrência estacional de *Doru luteipes* na cultura do milho em São Paulo, Brasil. Manejo Integrado de Plagas y Agroecología (Costa Rica) 70:46–49

[CR28] Haas F (2018) Biodiversity of Dermaptera. In: Foottit RG, Adler PH (eds) Insect biodiversity: science and society, 2nd edn. Wiley, pp 315–334

[CR29] Haas F (2021) Dermaptera. In: Rafael JA et al. Insetos do Brasil: Diversidade e taxonomia, 1st edn. Holos, Ribeirão Preto, pp 297–307

[CR30] Hanel A, Orpet RJ, Hilton R et al (2023) Turning a pest into a natural enemy: removing earwigs from stone fruit and releasing them in pome fruit enhances pest control. InSects 14:906. 10.3390/insects14120906 38132580 10.3390/insects14120906 PMC10743910

[CR31] Hanel A, Nottingham LB, Orpet RJ et al (2025) Evaluating trapping methods to increase earwig capture in temperate tree fruit crops. J Econ Entomol 118:551–560. 10.1093/jee/toaf018 39918400 10.1093/jee/toaf018

[CR32] Hehar G, Gries R, Gries G (2008) Re-analysis of pheromone-mediated aggregation behaviour of European earwigs. Can Entomol 140:674–681. 10.4039/n08-026

[CR33] Heleodoro RA (2024) Forficulidae in Catálogo Taxonômico da Fauna do Brasil. In: http://fauna.jbrj.gov.br/fauna/faunadobrasil/5787. Accessed 15 July 2025

[CR34] Hopkins H, Haas F, Deem LS (2022) Dermaptera species file. In: https://dermaptera.speciesfile.org/. Accessed 15 July 2025

[CR35] Jarvis KJ, Haas F, Whiting MF (2005) Phylogeny of earwigs (Insecta: Dermaptera) based on molecular and morphological evidence: reconsidering the classification of Dermaptera. Syst Entomol 30:442–453. 10.1111/j.1365-3113.2004.00276.x

[CR36] Jordan EO, Hefferan M (1966) Observations on the bionomics of *Euborellia annulipes* (Dermaptera: Labiduridae). Ann Entomol Soc Am 59:441–450

[CR37] Kocarek P, Dvorak L, Kirstova M (2015) *Euborellia** annulipes* (Dermaptera: Anisolabididae), a new alien earwig in Central European greenhouses: Potential pest or beneficial inhabitant? Appl Entomol Zool 50:201–206. 10.1007/s13355-015-0322-2

[CR38] Maggio DH, Rossetti VZ, Santos LMA et al (2022) A molecular marker to identify *Spodoptera frugiperda* (JE Smith) DNA in predators’ gut content. InSects 13:635–646. 10.3390/insects13070635 35886810 10.3390/insects13070635 PMC9319052

[CR39] Marucci RC, Souza IL, Silva LO et al (2019) Pollen as a component of the diet of *Doru luteipes* (Scudder, 1876) (Dermaptera: Forficulidade). Braz J Biol 79:584–588. 10.1590/1519-6984.184072 30365637 10.1590/1519-6984.184072

[CR40] McCormick A, Unsicker SB, Gershenzon J (2012) The specificity of herbivore-induced plant volatiles in attracting herbivore enemies. Trends Plant Sci 17:303–310. 10.1016/j.tplants.2012.03.012 22503606 10.1016/j.tplants.2012.03.012

[CR41] Meunier J (2024) The biology and social life of earwigs (Dermaptera). Annu Rev Entomol 69:259–276 37722682 10.1146/annurev-ento-013023-015632

[CR42] Mithöfer A, Boland W (2012) Plant defense against herbivores: chemical aspects. Annu Rev Plant Biol 63:431–450. 10.1146/annurev-arplant-042110-103854 22404468 10.1146/annurev-arplant-042110-103854

[CR43] Monteiro RC, Mound LA, Zucchi RA (1999) Thrips (Thysanoptera) as pests of plant production in Brazil. Rev Bras Entomol 43:163–171

[CR44] Moore AJ, Wilson P (1993) The evolution of sexually dimorphic earwig forceps: social interactions among adults of the toothed earwig, *Vostox apicedentatus*. Behav Ecol 4:40–48. 10.1093/beheco/4.1.40

[CR45] Nagoshi RN, Rosas-Garcia NM, Meagher RL et al (2015) Haplotype profile comparisons between *Spodoptera frugiperda* (Lepidoptera: Noctuidae) populations from Mexico with those from Puerto Rico, South America, and the United States and their implications to migratory behavior. J Econ Entomol 108:135–144. 10.1093/jee/tou044 26470113 10.1093/jee/tou044

[CR46] Naranjo-Guevara N, Peñaflor MFGV, Cabezas-Guerrero MF, Bento JMS (2017) Nocturnal herbivore-induced plant volatiles attract the generalist predatory earwig *Doru luteipes* Scudder. Naturwissenschaften 104:77. 10.1007/s00114-017-1498-9 28871442 10.1007/s00114-017-1498-9

[CR47] Nascimento DV, Bermúdez NC, de Oliveira GM et al (2025) Friend or foe? Revealing the omnivorous feeding behavior of the ring-legged earwig on brassicas. Arthropod-Plant Interact 19:62. 10.1007/s11829-025-10166-w

[CR48] Oliveira CM, Auad AM, Mendes SM, Frizzas MR (2014) Crop losses and the economic impact of insect pests on Brazilian agriculture. Crop Prot 56:50–54. 10.1016/j.cropro.2013.10.022

[CR49] Orpet RJ, Crowder DW, Jones VP (2019) Biology and management of European earwig in orchards and vineyards. J Integr Pest Manag 10:21. 10.1093/jipm/pmz019

[CR50] Orpet RJ, Curtiss RT, Catron KA et al (2025) Inoculation and conservation of the biocontrol agent European earwig in Washington pear orchards. Entomol Exp Appl 173:246–259. 10.1111/eea.13536

[CR51] Pacheco RC, Silva DD, Mendes SM et al (2023) How omnivory affects the survival and choices of earwig *Doru luteipes* (Scudder) (Dermaptera: Forficulidae)? Braz J Biol 83:e243890. 10.1590/1519-6984.243890 10.1590/1519-6984.243890 34133491

[CR52] Paredes-Sánchez FA, Rivera G, Bocanegra-García V et al (2021) Advances in control strategies against *Spodoptera frugiperda*. A review. Molecules 26:5587 34577058 10.3390/molecules26185587 PMC8471127

[CR53] Parra JRP, Coelho A (2022) Insect rearing techniques for biological control programs, a component of sustainable agriculture in Brazil. InSects 13:105. 10.3390/insects13010105 35055948 10.3390/insects13010105 PMC8778874

[CR54] Pasini A, Parra JRP, Lopes JM (2007) Dieta artificial para criação de Doru luteipes (Scudder) (Dermaptera: Forficulidae), predador da lagarta-do-cartucho do milho, Spodoptera frugiperda (J.E. Smith) (Lepidoptera: Noctuidae). Neotrop Entomol 36. 10.1590/S1519-566X2007000200020 10.1590/s1519-566x2007000200020 17607467

[CR55] Paula FF, Souza Gonçalves GA, Camargo NF et al (2026) Use of agricultural bioinputs and chemical inputs: implications for earwig diversity and pest control in soybean fields. Biol Control 212:105936. 10.1016/j.biocontrol.2025.105936

[CR56] Pedroso E do C, Otuka AK, Veiga ACP et al (2010) Consumo e desenvolvimento de *Doru luteipes* (Scudder) alimentado com ovos de *Plutella xylostella* (L.). Hortic Bras 28:672–675

[CR57] Perrin M, Delattre T, Borowiec N et al (2025) Influence of high temperatures on the European earwig *Forficula auricularia* s.l. and the parasitoid *Mastrus ridens*, two natural enemies of the codling moth *Cydia pomonella*. Biol Control 206:105802. 10.1016/j.biocontrol.2025.105802

[CR58] Picanço MC, Galvan TL, Galvão JCC et al (2003) Intensidades de perdas, ataque de insetos-praga e incidência de inimigos naturais em cultivares de milho em cultivo de safrinha. Cienc Agrotecnol 27:339–347. 10.1590/S1413-70542003000200013

[CR59] Pinto-Zevallos DM, Strapasson P, Zarbin PHG (2016) Herbivore-induced volatile organic compounds emitted by maize: electrophysiological responses in *Spodoptera frugiperda* females. Phytochem Lett 16:70–74. 10.1016/j.phytol.2016.03.005

[CR60] Quarrell SR, Corkrey R, Allen GR (2021) Cherry damage and the spatial distribution of European earwigs, (*Forficula auricularia* L.) in sweet cherry trees. Pest Manag Sci 77:159–167. 10.1002/ps.6003 33411365 10.1002/ps.6003

[CR61] Rankin SM, Palmer, JO (2009) Dermaptera. In: Encyclopedia of insects. Academic Press, pp 259-261. 10.1016/B978-0-12-374144-8.00079-5

[CR62] Rankin SM, Fox KM, Stotsky CE (1995) Physiological correlates to courtship, mating, ovarian development and maternal behaviour in the ring-legged earwig. Physiol Entomol 20:257–265

[CR63] Redoan ACM, Cruz I, Amâncio MB, da Silva CA, da Silva RB, Silva CRS (2014) Preferência alimentar de *Doru luteipes* a ovos de *Anagasta kuehniella*, *Spodoptera frugiperda *e *Helicoverpa armigera*. In: XXX Congresso Nacional de Milho e Sorgo, Salvador

[CR64] Reis LL, Oliveira LJ, Cruz I (1988) Biologia e potencial de *Doru luteipes* no controle de *Spodoptera frugiperda*. Pesqui Agropecu Bras 23:333–342

[CR65] Resende RC, Rodrigues-Silva AL, Pec M et al (2025) *Doru luteipes*: susceptibility to entomopathogenic fungi and the role of maternal care in the protection of offspring against infection. J Insect Behav 38:10–25. 10.1007/s10905-025-09874-1

[CR66] Ribeiro CI, César C, Coelho S, Rocha MS, Martins LO, Damasceno NC, Fernandes C, Souza S, Mendes SM (2017) Capacidade predatória de *Doru luteipes* e *Euborellia annulipes* sobre *Helicoverpa armigera*. In: Embrapa Agricultura Digital (CNPTIA) (ed) Seminário de Iniciação Científica PIBIC/BIC Júnior. Embrapa Milho e Sorgo, Sete Lagoas

[CR67] Rodrigues-Silva AL, Gualberto PL, Simão SD et al (2024) New perspective on the role of *Doru luteipes* as a predator of the fall armyworm: non-consumptive effects, predatory preference and functional response. J Appl Entomol. 10.1111/jen.13321

[CR68] Romero Sueldo GM, Virla EG (2017) Datos biológicos de *Doru luteipes* (Dermaptera: Forficulidae) en plantaciones de caña de azúcar y consumo de huevos de *Diatraea saccharalis* (Lepidoptera: Crambidae) en condiciones de laboratorio. Rev Soc Entomol Argent 68:359–363

[CR69] Romero Sueldo M, Bruzzone OA, Virla EG (2010) Characterization of the earwig, *Doru lineare*, as a predator of larvaeof the fall armyworm, Spodoptera *frugiperda*: A functional response study. Journal of Insect Science 10:38–47. https://doi.org/10.1673/031.010.3801 10.1673/031.010.3801 PMC3014733 20575739

[CR70] Romero Sueldo M, Dode M, Virla EG (2014) Depredación de *Doru luteipes* y *D. lineare* (Dermaptera: Forficulidae) sobre *Rhopalosiphum maidis* (Hemiptera: Aphididae) en condiciones de laboratorio. Acta Zool Lilloana 58:73–79

[CR71] Signoretti AGC, Peñaflor MFGV, Bento JMS (2012) Fall armyworm, *Spodoptera frugiperda* (J.E. Smith) (Lepidoptera: Noctuidae), female moths respond to herbivore-induced corn volatiles. Neotrop Entomol 41:22–26. 10.1007/s13744-011-0003-y 23950005 10.1007/s13744-011-0003-y

[CR72] Silva LP, Marucci RC (2025) Mating behavior and reproductive success in *Doru luteipes* (Dermaptera: Forficulidae). J Insect Behav 38:4. 10.1007/s10905-024-09867-6

[CR73] Silva GA, Picanço MC, Ferreira LR et al (2018) Yield losses in transgenic Cry1Ab and non-bt corn as assessed using a crop-life-table approach. J Econ Entomol 111:218–226. 10.1093/jee/tox346 29329399 10.1093/jee/tox346

[CR74] Silva LP, Souza IL, Marucci RC, Guzman-Martinez M (2023) *Doru luteipes* (Dermaptera: Forficulidae) and *Orius insidiosus* (Hemiptera: Anthocoridae) as nocturnal and diurnal predators of thrips. Neotrop Entomol 52:263–272. 10.1007/s13744-022-00982-7 35831705 10.1007/s13744-022-00982-7

[CR75] Souza CSF, Silveira LCP, Souza BHS et al (2021) Efficiency of biological control for fall armyworm resistant to the protein cry1f. Braz J Biol 81:154–163. 10.1590/1519-6984.224774 32159617 10.1590/1519-6984.224774

[CR76] Sujii ER, Beserra VA, Ribeiro PH et al (2007) Community of natural enemis and natural biological control of the aphid *Aphis gossypii* Glover (Hemiptera: Aphididae) and cotton leafworm *Alabama **argilácea* Hübner (Lepidoptera: Noctuidae) in the cotton crop. Arq Inst Biol (Sao Paulo) 74:329–336. 10.1590/1808-1657v74p3292007

